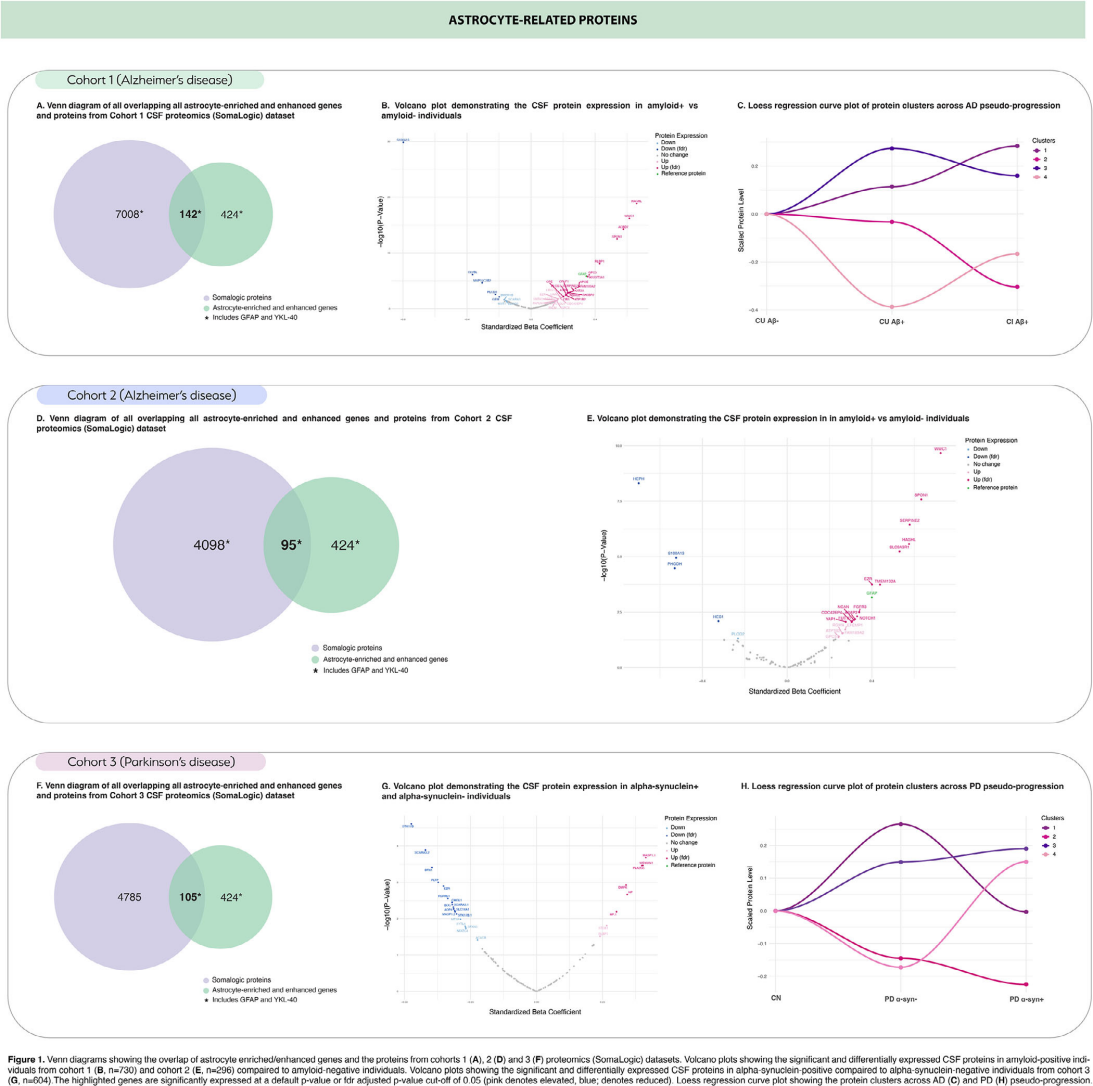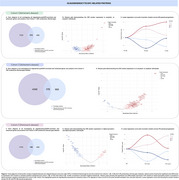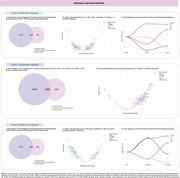# Novel CSF glial‐related biomarkers reflect Alzheimer's and Parkinson's pathological changes

**DOI:** 10.1002/alz70856_103748

**Published:** 2025-12-26

**Authors:** Luiza Santos Machado, Guilherme Povala, Ilaria Pola, Dzeneta Vizlin‐Hodzic, Pedro Rosa‐Neto, Eduardo R. Zimmer, Kaj Blennow, Henrik Zetterberg, Nicholas J. Ashton, Andrea L. Benedet

**Affiliations:** ^1^ University of Gothenburg, Gothenburg, VG, Sweden; ^2^ University of Pittsburgh, Pittsburgh, PA, USA; ^3^ Department of Psychiatry and Neurochemistry, Institute of Neuroscience and Physiology, The Sahlgrenska Academy, University of Gothenburg, Mölndal, Sweden; ^4^ University of Gothenburg, Gothenburg, Västra Götaland, Sweden; ^5^ McGill University, Montreal, QC, Canada; ^6^ Universidade Federal do Rio Grande do Sul, Porto Alegre, RS, Brazil; ^7^ Institute of Neuroscienace and Physiology, University of Gothenburg, Mölndal, Västra Götaland, Sweden; ^8^ Institute of Neuroscience and Physiology, Sahlgrenska Academy at the University of Gothenburg, Gothenburg, Sweden; ^9^ Banner Alzheimer's Institute, Phoenix, AZ, USA

## Abstract

**Background:**

Glial cells play important roles in the pathophysiology of neurodegenerative diseases, including Alzheimer's disease (AD) and Parkinson's disease (PD). Glial dysfunction is thought to contribute to neurodegeneration progression, but a few cerebrospinal fluid (CSF) biomarkers effectively capture these changes. Identifying novel glial‐derived CSF biomarkers may improve our understanding of AD and PD pathology and aid in disease monitoring.

**Method:**

We selected astrocyte‐, oligodendrocyte‐, oligodendrocyte precursor cell (OPC)‐, and microglia‐enriched and enhanced genes from single‐cell and single‐nucleus transcriptomic datasets. These gene lists were cross‐referenced with CSF proteomic data (SomaLogic) from two AD cohorts (cohorts 1 and 2) and a PD cohort (cohort 3) (Figures 1‐3ADF). Linear regression models were used to assess differential protein expression in CSF between cognitively unimpaired (CU) and cognitively impaired (CI) individuals, as well as between amyloid‐beta (Aβ)‐positive and alpha‐synuclein (α‐syn)‐positive individuals, within each glial cell type. In cohorts 1 and 3, differentially abundant proteins were clustered based on their pseudo‐progression across CU and CI individuals, further stratified by Aβ (pTau181/Aβ42 ratio cut‐off=0.028) or α‐syn status. In a subset of cohort 1, voxelwise analyses examined associations between protein cluster averages and [^18^F]Florbetapir‐PET amyloid imaging, adjusting for age and sex. Random field theory (RFT) was applied for multiple comparison correction in imaging analyses.

**Result:**

We identified 47 astrocyte‐, 94 oligodendrocyte/OPC‐, and 94 microglia‐related proteins that were significantly altered in CI individuals compared to CU. In Aβ+ individuals, 52 astrocyte‐ (Figure 1BE), 99 oligodendrocyte/OPC‐ (Figure 2BE), and 117 microglia‐related proteins (Figure 3BE) were differentially expressed. Similarly, in α‐syn+ individuals, 143 glial proteins were altered across astrocytes, oligodendrocytes/OPCs, and microglia (Figures 1‐3G). Clustering analysis categorized proteins into four distinct groups per cell type and cohort (Figures 1‐3CH). In cohort 1, voxelwise analyses demonstrated that at least one cluster per cell type presented an association with [^18^F]Florbetapir‐PET imaging, particularly in cortical gray matter (data not shown).

**Conclusion:**

This study identified 485 glial‐related CSF proteins altered in AD or PD. We also uncovered distinct glial protein clusters across AD pseudo‐progression and their association with Aβ burden in the brain, highlighting potential glial biomarkers for neurodegenerative disease progression. These findings can help uncover the glial heterogeneity present in AD and PD.